# The Effect of Bacteria Modulation with Probiotic Consumption in Neurodegeneration During Aging: A Narrative Review of the Literature

**DOI:** 10.3390/diseases13100317

**Published:** 2025-09-26

**Authors:** Nayeli Valdez Gayosso, Arianna Omaña Covarrubias, Ana Teresa Nez Castro, Lydia López Pontigo, María del Refugio Acuña Gurrola, Bertha Maribel Pimentel Pérez

**Affiliations:** 1 Department of Nutrition, School of Medical Science, Autonomous University of the State of Hidalgo, Pachuca 42039, Mexico; va196511@uaeh.edu.mx (N.V.G.); teresa_nez@uaeh.edu.mx (A.T.N.C.); 2 Department of Gerontology, School of Medical Science, Autonomous University of the State of Hidalgo, Pachuca 42039, Mexico; lydial@uaeh.edu.mx (L.L.P.); maria_acuna@uaeh.edu.mx (M.d.R.A.G.); bertha_pimentel@uaeh.edu.mx (B.M.P.P.)

**Keywords:** aging, neurodegeneration, intestinal microbiota, probiotics

## Abstract

Aging is the result of the accumulation of a great variety of molecular and cellular damage over time. During aging, the brain undergoes changes and diseases such as depression, dementia, anxiety, Alzheimer’s, delirium, behavioral disorders and aggression, and prolonged mourning, among others, appear. The gut–brain axis suggests that the gut and the brain have a bidirectional communication, so it is important to maintain proper intestinal health to strengthen the neurological changes of this age group. The intestinal microbiota is a dynamic and highly complex ecosystem of microorganisms residing in the gastrointestinal tract. The bidirectional and dynamic communication between the homeostatic systems, such as the endocrine and immune systems, as well as the nervous system, allow us to face problems associated with several diseases. Probiotics are defined as non-pathogenic live microorganisms that provide beneficial effects to the organism and participate in the prevention and treatment of diseases, which is the reason why it is important to promote interventions that keep intestinal microbiota in eubiosis (microbiota balance). The concentration and balance of the intestinal microbiota depend on several conditions, such as diet, antibiotic consumption, and lifestyle, to mentioned a few. However, interventions with probiotics have shown improvements in both cognitive function and processes that promote neurodegeneration. It is such that the research has been directed on designing strategies that improve not only oral bioavailability but also intestinal adhesion and retention, to clarify the frequency and dosage that should be consumed.

## 1. Introduction

Old age begins at the age of 60 and is made up of the population that is in the last stage of their life. It is characterized by the presence of physiological changes characteristic of age, in addition to the aggravation of complications, mainly of non-communicable diseases. On the other hand, aging is also defined as a continuous, multifaceted and irreversible process that begins at birth and ends with death, consisting of a series of psycho-social, physical, and physiological changes [1].

It is worth mentioning that the world population is undergoing an aging process never seen before. The World Health Organization (WHO) reported that, in 2021, there were 761 million people over 65 years of age and by 2050 there will be 1600 million [1]. This population increase has been associated with a decrease in mortality due to scientific, technological, educational, and social developments, that have contributed to an increase in life expectancy. However, as we add more years to live, health needs to also become more specific. For this reason, promoting the importance of implementing healthy aging has been sought [1,2].

Current evidence indicates that aging is the main risk factor for neurodegeneration, that is understood as the progressive process of neuronal damage that leads to the loss of neuronal function and frequently to neuronal death [2]. Although it can develop for a variety of reasons, many pathologies of the nervous system can cause neurodegeneration, as well as metabolic disorders, infections, autoimmune diseases, and various toxins. In this sense, neurodegenerative diseases usually present in middle or advanced age and are characterized by a cumulative deterioration of different nervous system functions which hasten the death of the affected individuals due to the decrease in the capacity for cell renewal and repair [2,3]. As mentioned above, scientific evidence has described several mechanisms and factors that promote neurodegeneration, the intestinal microbiota being (IM) one of the most studied currently [3].

Since dysbiosis (an imbalance in the composition of the microbiota), has been associated with some central nervous system (CNS) disorders, it suggests a close relationship between intestinal health and brain function [3]. Thus, in the quest to understand the mechanism by which dysbiosis alters brain function, it has been assumed that it is through the gut–brain axis, the bidirectional communication network between the gut and the brain, which mainly includes neuroendocrine, neural, and immune signaling pathways [4].

Based on the above, research has focused on designing interventions that promote the modulation of the IM to contribute to the improvement of the gut–brain axis. Among the proposed interventions is probiotic supplementation, due to their systemic anti-inflammatory effect, in addition to the fact that they produce short-chain fatty acids and tryptophan, substances that can indirectly improve CNS functions [5].

While it is true that the relationship between the brain and gut in the development of neurodegenerative diseases has been becoming more concrete, it is crucial to understand the impact of bidirectional communication between the IM and the brain in maintaining homeostasis, in addition to determining the impact of probiotic consumption on the treatment of behavioral and cognitive functioning problems [3,4].

Due to the above, this paper aims to describe the relationship between aging and neurodegeneration mediated by IM. It also aims to develop current knowledge on the impact of probiotic consumption on IM modulation as preventive and control therapy to neurodegeneration during aging.

The search strategy for this narrative review was designed to encompass a wide range of studies examining the changes that result from aging in the elderly, as well as the effects of probiotic consumption for the prevention or delay of neurodegeneration.

## 2. Material and Methods

A systematic search was conducted using the PubMed electronic database (https://pubmed.ncbi.nlm.nih.gov accessed on 13 September 2025), focusing on the literature published between 2015 and 2025, considering as inclusion criteria publications in which changes due to aging were analyzed, including neurodegeneration processes in people over 60 years of age, without restriction by sex or demographic data, in addition to the consumption of probiotics and their effects on the IM. In vitro analyses were excluded, as well as animal studies, and the selection was limited to complete articles.

## 3. Aging and the Nervous System

The “chronological age” determines the aging process, starting at 60 and 65 years of age. On the other hand, it is known that neuron aging begins around 30s. It is characterized by the decrease in the ability to respond to stress and maintain homeostatic and metabolic regulation [6]. This generates a progressive loss in its functioning that makes the human being vulnerable to neurodegenerative diseases development. Because the brain seems to be particularly sensitive to the aging process, it turns out to be the main risk factor in the onset of neurodegenerative diseases [6,7].

The changes that most frequently occur in the brain as a consequence of aging include decreased brain weight and volume, cortical atrophy, loss of cortical neurons and some subcortical nuclei, and increased lipofuscin granules in neurons and glia, as well as hypertrophic changes in astrocyte glia [6]. On the other hand, the cells of the nervous system also present changes in their components in relation to aging, such as increased oxidative stress, and accumulation of damage in proteins, lipids, and nucleic acids [8].

Furthermore, during aging, a decline in cognitive performance has been observed, mainly in aspects of working, such as spatial and episodic memory, while emotional, automatic, and autobiographical memory do not usually present this behavior. These modifications are associated with neuroanatomical changes, such as a decrease in gray matter volume, not associated with pathological conditions. A reduction in white matter density has also been observed, and an increase in the number of white matter lesions. Lifestyle factors, such as a lack of cognitive apathy, have been identified as significantly influencing the development of neurodegenerative disorders [9].

Therefore, it is likely that changes and deterioration of other systems such as the gastrointestinal or endocrine systems may accelerate the brain aging process. These facts have generated a significant increase in the prevalence of diseases associated with aging, especially dementias [6,10].

Likewise, aging is characterized by a gradual decline in cognitive processes such as executive functions, episodic memory, working memory, and brain processing speed. Gollan et al. conducted a study at the Alzheimer’s Disease Research Center (ADRC) on 100 Hispanic participants with dementia and without dementia, performing clinical diagnosis through medical examinations and cognitive tests (MMSE Mini-Mental State Examination, DRS-R-98 Delirium Rating Scale- Revised-98), as well as their educational level. This ability to possess sufficient mental stock that can prevent harmful effects of aging on our cognition is known as “cognitive reserve” [11]. Cognitive reserve leads to the development and increase in the number of synapses that are established between neurons and improves various aspects of the brain, such as increased metabolites to the brain, and improved blood supply and vascularization, along with the associated increase in cerebral oxygenation capacity [12].

Mortimer et al. conducted a study to examine the prevalence of dementia associated with having a smaller brain, less education, or both characteristics in Catholic sisters of the School Sisters of Notre Dame congregation who resided in communities in the Midwest, Eastern, and Southern United States. In this study, the brains of sixty deceased nuns were evaluated to determine whether they met the neuropathological criteria for Alzheimer’s disease. They observed that people with head circumferences in the lower two tertiles and with less than 16 years of education were four times more likely to have dementia. However, no increased risk was observed among people with a large head circumference and less than 16 years of education or among people with a small head circumference and 16 or more years of education. So, the additional brain reserve provided by a larger brain or a higher education might be sufficient to substantially reduce the likelihood of dementia [13].

Likewise, in China, Jiang et al. conducted a randomized clinical trial in which they evaluated whether preoperative cognitive training reduces the incidence of delirium in patients who underwent coronary artery bypass graft surgery, referring to the fact that alterations in attention, short-term memory, and visuospatial processing are associated with postoperative delirium and that cognitive reserve may be a potentially modifiable protective factor to prevent the development of postoperative delirium and postoperative cognitive dysfunction (POCD). The results reported in this study indicated that patients who received cognitive training were 57% less likely to develop delirium than those who received routine care and those with more education. It is worth mentioning that this cognitive reserve develops during childhood and early adulthood and should be stimulated to be maintained in old age [14].

Thus, aging has been associated with a loss in the number and/or activity of neural stem cells, which could explain the decrease in brain function. Therefore, the design of interventions focused on the prevention of stem cell deterioration could increase human cognitive health [15]. Several studies have suggested that the IM is a relevant factor for proper brain function and that its imbalance (dysbiosis) is associated with the development of neurodegenerative diseases [3].

### 3.1. Intestinal Microbiota

Microbiota refers to the community of living organisms residing in a given environment. The microbiota is distributed in different cavities of the human body, from the mouth, in the acoustic meatus, the pharyngeal cavity, the surface of the dermal layer, and the digestive tract, among others, the IM being one of the densest as it includes all species of microorganisms residing in the gastrointestinal tract. This microbiota is indispensable for various metabolic functions, such as the development of immunity and nutrition [3,16].

Even though colonization of the IM begins in uterine life, the composition, diversity, and metabolism changes throughout the different stages of life [16,17]. The development of IM begins before birth, because of the exposure of the fetus to maternal microbial metabolites and bacterial communities from the skin, vaginal, oral cavity, and gastrointestinal tract. At birth, there is a significant transfer of the microbiota from mother to baby, which is determined by the type of delivery. Subsequently, the type of feeding determines the transfer of microorganisms, maintaining a stable colonization until the introduction of complementary feeding, at which point IM dynamically diversify until the first five years of age. From this moment and throughout adulthood, colonization remains relatively stable until the age of 60, at which point its diversity decreases again [17,18,19].

During aging, the IM changes to a less healthy one, with an increase in *E. coli* and other proteobacteria, along with a decrease in beneficial anaerobes such as *Bifidobacteria* and *Bacteroides*, and a decrease in the *Firmicutes/Bacteroidetes* ratio. On the other hand, an increase in *Akkermansia* has been found in centenarians, which has been related to longevity [20].

Studies focused on the analysis of intestinal bacterial communities in older adults have determined that the most predominant species are *Bacteroides*, *Prevotella*, and *Ruminococcus*, regardless of nationality, sex, age, or BMI. Likewise, a lower proportion of *Firmicutes* and *Bacteroidetes* has been reported compared to younger adults, in addition to a lower count of short-chain fatty acids (SCFA)-producing species. All of these modifications are even associated with the immunosenescence and fragility characteristics of aging [21].

Thus, metagenomic studies have identified bacterial species as potential biomarkers of aging, such as *Bacteroides*, *Eubacterium*, and *Bifidobacterium*. Likewise, the presence of *Akkermansia muciniphila* has been associated with improved health. Thus, alterations in the composition, diversity, and functionality of the immune system are closely related to conditions associated with aging, such as immunosynthesis and inflammaging, which in turn reduce longevity and contribute to increased morbidity and mortality [22].

The bacterial colonization of the human intestine begins at birth, and subsequently depends on intrinsic factors such as genetics and physiological state, while extrinsic factors considered are diet, bacterial exposure during childhood, stress levels, puberal development, antibiotic consumption, and aging, and these conditions can lead to a dysbiosis, a state related to a wide variety of health conditions [23].

In addition, nine characteristics of aging have been identified as follows: genomic instability, telomere wear, epigenetic alterations, loss of proteostasis, mitochondrial dysfunction, cellular senescence, deregulated nutrient detection, stem cell exhaustion, and altered intercellular communication, which have been related to changes in the microbiome [24].

Thus, aging has been associated with the progressive alteration of the physiological balance between the host and the commensal IM, a situation defined as dysbiosis. The above is due to a greater diversity in the IM being associated with a better health state, characteristic of youth, while a low microbial diversity has been associated with the disease state, typical of aging [24,25].

It is worth mentioning that changes in bacterial diversity during aging have been linked to changes in diet, immunosenescence, and exposure to various drugs. In addition, intestinal dysbiosis contributes to the development of a wide variety of diseases, which consequently decreases healthy aging [21]. Therefore, recent information suggests that maintaining a “young” or “healthy” IM may contribute to the maintenance or control of immunosenescence [22]. Although genera such as *Akkermansia*, *Bifidobacterium*, and *Christensenellaceae* have been identified as being related to longevity, there is a common tendency to accumulate mostly potentially proinflammatory metabolites [18].

Hence the importance of maintaining an eubiosis, because it conditions homeostatic systems such as the endocrine and immune systems but also the nervous system. To maintain this eubiosis, it is important to maintain a healthy lifestyle and maintain the consumption of probiotics, which will ensure a healthy longevity [26].

### 3.2. Gut–Brain Axis

The gut–brain axis suggests that the gut and the brain have a bidirectional communication, maintaining communication through the modulation of the immune system, the vagus nerve, the enteric nervous system, the neuroendocrine system and the circulatory system, a process that is carried out through the synthesis of neuroactive substances, metabolites, and hormones. Due to the above, communication between the organs occurs and can influence the function of the other, so it is important to maintain proper intestinal health in order to strengthen the cognitive reserve and thus be able to delay the neurological changes characteristic of this age group [27,28].

The neural pathways of bidirectional gut–brain communications are intuitive. The vagus nerve is the tenth cranial nerve, from which the hepatic and celiac branches innervate the intestine which in turn transmits neurohormones, among which cholecystokinin (CCK), glucagon-like peptide-1 (GLP-1), peptide YY (PYY), serotonin, etc., stand out. These hormones propagate to afferent terminals and bind to their receptors, fulfilling different functions among them, such as the regulation of food intake and digestion, either directly in the brain or indirectly through the vago-cerebral pathway [29].

For this reason, intestinal dysbiosis has currently been considered a factor that promotes neurodegeneration, since it has been observed that in adults with Alzheimer’s diseases, there is a significant alteration in the structure and composition of the IM, a situation that can increase intestinal permeability and induce inflammation. In addition, in older adults with neurodegenerative diseases, a reduced intestinal bacterial biodiversity has been observed [30].

IM dysregulation and subsequent neuroinflammation may play a crucial role in the pathogenesis of neurodegenerative diseases. IM modulates intestinal permeability and various immune functions by secreting toxins and fatty acids; thus, its disruption leads to oxidative stress, inflammation, altered blood–brain barrier (BBB), immune system activity, neurodibrial tangles, and Aβ plaques followed by neurodegeneration [30].

It is such that dysbiosis promotes the decrease of the SCFA and increase of harmful microbial components, as the lipopolysaccharide and inflammatory cytokines (such as interleukin-6 and tumor necrosis factor alpha), which in turn increases the permeability of the BBB and promotes neuroinflammation [31].

The reason why the diversity and composition of intestinal bacteria determine the abundance of microbiota-derived metabolites, neurotransmitters, and SCFA such as butyrate, propionate, and acetate is because the imbalance of these intestinal bacteria alters the aforementioned processes and results in an inadequate intestinal environment. This profile generates an environment of signaling molecules that can ultimately communicate with the brain through neural communication via the vagus nerve, and endocrine signaling involving the hypothalamic–pituitary–adrenal (HPA) axis and the immune system, which as a means of preservation and defense, increases cytokine secretion, modulating brain function, behavior, and cognition [14,32]. It has been shown that IM bacteria can produce different neurotransmitters, such as gamma-aminobutyric acid (GABA), which is produced by bacteria of the genus *Lactobacillus* and *Bifidobacterium*. This inhibitory neurotransmitter is the most important neurotransmitter in the central nervous system since dysfunction of the GABAergic system is implicated in the pathophysiology of several mental health conditions such as depression, anxiety, autism, and schizophrenia [5].

This is based on the fact that the symbiotic relationship between the host and the IM occurs through bidirectional signaling between bacteria and the CNS, which includes endocrine and immune pathways, but also mood, emotional, cognitive, and even habitus, as well as environmental interaction, where the communication pathways of the microbiota-gut–brain axis are schematized [33].

It is worth mentioning that lifestyle, and modification in eating habits, as well as the environment, can cause changes in the IM composition, which subsequently affect the intestine–brain axis. The relationship between IM and the environment is observed with greater evidence in people with scarce resources and poor sanitary conditions, especially in those who are constantly exposed to contaminated water and food, who are also characterized by anemia and impaired brain development, as well as epithelial affection with loss of enzymes and decrease in the functional surface of the intestine that causes poor digestion and malabsorption, in addition to alterations in the balance of the IM [34].

Among the changes in dietary habits that alter the composition of the IM, diets rich in fat or carbohydrates can be observed. Changes in temperature significantly influence the composition of IM and contribute to alterations in neurotransmitter levels in the stool, blood, and central nervous system [35].

### 3.3. Intestinal Microbiota and Neurodegeneration

There is growing evidence about the role of IM in neurodegenerative diseases, primarily due to microglial activation and neuroinflammation, characteristics of neurodegeneration. Because current treatment for neurodegenerative disease is based on controlling symptoms and disease progression, research has sought to design approaches beyond these types of interventions [28,31].

As previously reviewed, neurodegeneration is strongly influenced by the relationship between the gut–brain axis, the IM, and its metabolites, such as SCFA, bile acids, and tryptophan metabolites; which is why the modulation of IM for the production of metabolites that mitigate neurodegenerative processes is an emerging area of interest [31,36].

Dysbiosis can lead to increased intestinal permeability, which allows the passage of proinflammatory cytokines and microbial products into the bloodstream, which in turn generates systemic inflammation that, when extended to the central nervous system, can lead to neuroinflammation, neuronal damage, and cognitive decline, accelerating neurodegeneration [36,37,38].

Another factor that may play a role in neuroinflammation is the involvement of IM in the metabolism of several neurotransmitters responsible of mood regulation, cognition, and behavior, such as serotonine, dopamine, and acetylcholine, which are involved in the development of neuropsychiatric disorders such as depression, anxiety, and Parkinson’s disease. Likewise, altered levels of neurotransmitters can negatively affect not only the formation and plasticity of brain synapses, but also the enteric nervous system. For this reason, the relationship between dysbiosis and neurotransmitter metabolism would prove to be a crucial therapeutic approach for diseases such as depression, Parkinson’s disease, and Alzheimer’s [31,37].

Similarly, SCFA-producing bacteria are essential for maintaining the intestinal barrier and modulating immune homeostasis. Once butyrate, propionate, and acetate cross the BBB, they enhance the expression of tight junction proteins, which prevents the influx of neurotoxic substances into the brain. For this reason, these fatty acids play an important role in controlling neuroinflammation, promoting neurogenesis, and contributing to overall neurofunction and neurotrophic factors [38,39].

Among the IM modifications related to neurodegeneration, the modification in *F/B* ratio has been identified, particularly due to the decrease in *Firmicutes* and the increase in *Bacteroidetes*. On the other hand, the increase in the relative abundance of *Ruminococcaceae*, *Enterococcaceae*, and *Lactobacillaceae*, as well as the decrease in *Lanchnospiraceae*, *Bacteroidaceae*, and *Veillonellaceae*, have been observed in patients with Alzheimer’s disease [22]. Likewise, the reduction in the concentration of *Faecalibacterium* sp., *Bifidobacterium* sp., and *Akkermansia* sp., can increase neuronal injury, inflammation, and oxidative stress, leading to neurocognitive dysfunction [31].

Similarly, the reduction of *Bifidobacterium* and *Lactobacillus* with the increase of intestinal concentrations of *proteobacterias* and *bacteroids*, are related to the increase of lipopolysaccharides, a bacterial endotoxin that is characterized by disrupting the function of the blood–brain barrier [31,37].

On the other hand, the *Verrucomicrobia* phylum was found to be increased in Parkinson’s disease and Alzheimer’s disease, so research has correlated it with increased levels of LPS and consequently of proinflammatory factors such as TNFα, IL6, and C-reactive protein (CRP). A similar situation is observed with *Collinsella*, which is positively associated with neurodegeneration because it induces the degeneration of dopaminergic b¿neurons, which disrupts physiological maintenance and cell death [38].

Due to the above, the importance of maintaining a balanced IM, in which *Lactobacillus* and *Bifidobacteria* predominate, is highlighted, since the metabolites produced by these are considered an important pillar in the prevention and control of neurodegeneration [36].

### 3.4. Probiotic Supplementation and Neurodegeneration

Due to the growing field of research on the impact of dysbiosis on neurodegeneration, human clinical trials have focused on therapeutic modulation of IM, by dietetical interventions and consumption of probiotics, herbal medicines, probiotics, and symbiotics. This is conducted with the aim of promoting the production of SCFAs that have a neuroprotective role, by reversing dysbiosis [25,39].

Probiotics are non-pathogenic and generally safe microorganisms that are marketed and consumed as dietary supplements or functional foods because they promote probiosis mechanisms that include modulation of microbial communities, pathogen suppression, immunomodulation, epithelial cell stimulation, or intestinal barrier integrity. Due to the above, the administration of probiotics reinforces the hypothesis of the bidirectional relationship of the gut–brain axis for the prevention of intestinal dysbiosis [40].

The mechanism of action of probiotics is mainly based on preventing colonization and invasion by pathogenic microorganisms, improving the barrier function of the gastrointestinal tract, modulating the immune system, and reducing the proinflammatory response [40]. According to Florencia-Martinez et al. 2022 [41], among the most common mechanisms of action of probiotics are resistance to colonization through competition with pathogens, synthesis of short-chain fatty acids, regulation of the microbiota and enterocyte regeneration. Among frequent mechanisms include vitamin and mineral synthesis, strengthening of intestinal barrier, metabolism of bile salts and avoid calcium-based collectors, while rare mechanisms include endocrinological, immunological and neurological effects [40].

The goal of IM modulation therapies is to rebuild a dysbiotic microbiota into a microbiota in eubiosis. Probiotics are usually composed of combinations of the genera *Lactobacillus* and *Bifidobacteria*, which suppress inflammation and modulate the immune system by preventing the induction of the cytokine IL-8 in the human colonic epithelium, as well as reducing intestinal permeability and inhibiting endotoxemia [41,42].

This, due to positive changes in intestinal metabolites, such as SCFA, plays a crucial role in neuroreactivity by reducing neuroinflammation. This also improves tryptophan production, which in turn increases serotonine production [38].

As part of the therapeutic promises, it has been identified that oral supplementation with *Bifidobacterium* increases the concentration of lymphocytes, improving the activity of natural killer cells, phagocytosis in peripheral cells, and the concentration of neutrophils, contributing to the decrease of cellular immunosenescence [22]. Other trials have identified that multi-strain probiotics (composed of *Bifidobacterium lactis*, *Lactobacillus bulgaricus*, *Streptococcus thermophilus*, and *Lactobacillus lactis*), increase the concentrations of fatty acids that maintain adequate brain function, learning memory, and neurogenesis [29].

Similarly, it has been shown that an imbalance in SCFA production is associated with the development of diseases that promote neurodegeneration, such as Alzheimer’s and Parkinson’s. The reason why is because of the recommended supplementation with SCFA-producing bacteria such as *Lactobacillus, Bifidobacterium*, and *Clostridium*, particularly *Lactobacillus* and *Bifidobacterium*, which are key strains in the homeostasis of the gut–brain axis, due to their influence on reducing systemic inflammation and strengthening tight junctions. On the other hand, *Lactobacillus rhamnosus* modulates proinflammatory cytokines (TNFα and IL-6), and it directly strengthens the function of the BBB, as well as *Bifidobacterium longum* and *Bifidobacterium breve*, by restoring SCFA levels, reducing oxidative stress and glial activation. Likewise, it has been shown that the joint administration of *B. bifidum, L. acidophilus*, and *B. longum* in doses of 2 × 10^9^ CFU/day and selenium supplements, significantly improved cognitive performance [37,43].

Tian et al. 2022 conducted a double-blind, randomized controlled trial, where they studied Major Depressive Disorder (MDD) in patients over 18 years old without restrictions in the use of antidepressants, where they investigated possible psychotropic effects of a combined probiotic intervention of three strains *Bifidobacterium breve* CCFM1025, *Bifidobacterium longum* CCFM687, and *Pediococcus acidilactici* CCFM6432, evidencing a significant reduction in the depression indexes and significantly improving the gastrointestinal functions of the patients. Referring to that, the mechanisms possibly correlated with the modulation of hormones related to the HPA axis, brain serotonergic systems, and neuronal plasticity [44].

On the other hand, Schaub et al. 2022 tested the potential of a short-term high-dose probiotic supplementation as a complementary treatment for depression, where the main finding was an evident improvement of depressive symptoms after probiotic supplementation. Functional magnetic resonance imaging (fMRI) showed the brain structure called *putamen*, which is affected by depression, after the consumption of probiotics for 4 weeks during its activation. This postulates that *putamen* hyperactivity evoked by negative emotional stimuli contributes to the negativity biases found in patients with MDD, who perceive emotionally neutral faces as emotionally negative faces. It is worth noting the importance of compliance during probiotic supplementation as it is equally important as general antidepressant therapy, so it is important to consider the choice of probiotic formulation and dosage [45].

Another study on probiotic supplementation was conducted by Aljumaah et al., who performed a randomized, placebo-controlled, double-blind trial, in which the impact of *L. rhamnosus GG* supplementation on cognitive functions was evaluated in healthy adults aged 52–75 years for 3 months. The neuropsychological assessment consisted of completing the computerized NIH Toolbox for the Assessment of Neurological and Behavioral Function Cognition, which evaluates different cognitive domains. Among the results, the improvement in neuropsychological tests was observed during the three months of follow-up with probiotic supplementation. Thus, it was possible to demonstrate the existence of an association between IM and cognitive performance aimed at achieving successful aging and maintaining functionality and independence [46].

Asaoka et al. conducted a double-blind, randomized, controlled trial to study the effect of *Bifidobacterium breve* MCC1274 (A1) on improving cognition and preventing brain atrophy in elderly patients aged 65–88 years with mild cognitive impairment for 24 weeks, for which cognitive functions were assessed using the Advanced Driving Assistance Systems (ADAS-Jcog) and Mini-Mental State Examination (MMSE) tests, in addition to an MRI to determine changes in brain atrophy using the Voxel-based Voxel-Specific Regional Analysis System for Alzheimer’s Disease (VSRAD). In the results, test scores were obtained at baseline at week 8, 16, and 24, in which it was observed that scores were uniformly improving and as for VSRAD data, probiotic supplementation suppressed the progression of brain atrophy and therefore is a practical approach for the prevention of cognitive decline in subjects with mild cognitive impairment [47].

While knowledge in the field of probiotic supplementation and its impact on neurodegeneration continues to be elucidated, it is known that metabolites such as SCFA are important in maintaining adequate brain function, since they actively participate in neurogenesis. In addition, SCFA influences the maintenance of intestinal permeability, a condition that is associated with the appearance of disorders such as stress or depression [29].

On the other hand, the microbiota also contributes to modulating the expression of brain-derived neurotrophic factor (BDNF), which in turn promotes the survival of existing neurons as well as the growth of new neurons. Likewise, IM also participate in the production and modulation of neurotransmitters and neuromodulatory compounds such as GABA, serotonin, dopamine, acetylcholine, and noradrenaline [29].

Furthermore, the intestinal microorganisms induce the synthesis of SCFS, B12 vitamin, and neurotransmitters such as, serotonin, catecholamines, glutamate, and GABA, and neuromodulatory metabolites that can affect a person’s health [48].

As a result of bacterial translocation, a characteristic of the last stage of life, a series of systemic inflammatory responses are triggered and promote inflammation of the central nervous system and neurodegeneration. This is the reason why the microbiota–gut–brain axis represents a significant modulator in improving the development and progression of neurodegenerative diseases [28].

As part of clinical therapies for neurodegenerative diseases, those are considered based on bacterial modulation through the intake of prebiotics, probiotics, and/or dietary interventions for the control of symptoms and the development of pathological conditions. Particularly, the aim of probiotic consumption is to introduce specific microbial strains that promote the production of metabolites with antioxidant and anti-inflammatory properties [28,48,49].

Based on this fact, clinical trials in humans have shown that supplementation with probiotics such as *Escherichia coli*, *Lactiplantibacillus plantarum*, and *Bifidobacterium pseudocatenulatum* improve function and prevent cognitive decline after 12 weeks of follow-up [49]. On the other hand, research has shown that supplementation with *Bifidobacterium breve* promises to be an effective intervention to improve immediate, delayed, and special memory functions after 4 months of intervention. In addition, supplementation with *Lactiplantibacillus plantarum*, from fermented soy, improve the cognitive composite score of older people after 12 weeks of intervention, and other similar results were obtained with supplementation of *Lactobacillus* and *Lactobacillus rhamnosus GG* [50].

On the other hand, some studies do not show a direct improvement in cognitive function but they do influence the improvement of metabolic factors associated with cognition, such as insulin sensitivity. Such is the case with the supplementation of bacteria such as *Akkermansia muciniphila*, *Lactobacillus acidophilus*, *Lactobacillus casei*, *Bifidobacterium bifidum*, and *Bifidobacterium fermentum*, also after 12 weeks of supplementation [50].

The progress in research has been such that today, as a result of knowledge about IM and the development of new technologies, next generation probiotics (NGP) have been formulated, which go beyond traditional strains such as *A. muciniphila*, *Feacalibacterium preausnitzii*, *Roseburia intestinalis*, *and Bacteroides fragilis*, and these contribute to the maintenance of epithelial and endothelial integrity, enhance the function of the central nervous system barrier, maintain a considerable anti-inflammatory effect, and restore the balance of neurotransmitters, which makes them powerful neuroprotectors [28,37].

According to the review by Sheng et al., 2024, the supplementation of bacteria such as *Bifidobacterium breve* A1, *Lactiplantibacillus plantarum*, *Lactobacillus acidophilus*, *Lactobacillus casei*, *Bifidobacterium bifidum*, *Lactobacillus fermentum*, *Lactobacillus rhamnosus*, *Bifidobacterium longum*, *Lactobacillus lactis*, *Bifidobacterium infantile*, and *Lactobacillus reuteri*, in doses of 1–2 × 10^10^ UFC/day, improves cognitive function. In addition, a reduction in serum C-reactive protein levels (inflammation indicator) and plasma malondialdehyde (oxidative stress indicator) was observed. Furthermore, it improves insulin metabolism, triglyceride levels, and improves intestinal discharge and intestinal transit time [28].

In addition to the effects on cognitive function, supplementation with a mixture of five strains of *Lactobacillus* with five strains of *Enterococcus*, showed beneficial effects on the damage caused by high-fat diets, such as intestinal permeability, metabolism, immunity, and physical deterioration [50].

## 4. Conclusions

Several mechanisms have been described to explain the interaction between IM and brain function, this being a study of the gut–brain axis through solid evidence. Therefore, promoting the maintenance of a “young” or “healthy” IM is gaining ground as a promising intervention strategy. This is the reason why the therapeutic modulation of MI that contribute to the prevention and control of neurodegeneration has been increasing. The intervention with probiotics has a positive impact on cognitive and mental functions in the whole population and with special interest in the elderly.

The results of the studies analyzed show that probiotics have beneficial properties for health and nutritional status. Despite this, more studies are required to strengthen the implementation of probiotics for human use, especially to clarify the frequency and dosage that should be consumed, because of the variety of test results according to the supplementation conditions.

## 5. Future Orientations

The present research is intended to be a background for future research related to the intestinal microbiota, especially related to the benefits in the elderly, and thus achieve a benefit in their nutritional status. Also, it allows us to synthesize current knowledge regarding the effect and recommendations for clinical intervention with probiotics. However, future lines of research on probiotics aim to develop more specific and effective indications in the treatment of different clinical conditions, including the use of natural probiotics such as mead and other fermented beverages.

## Abbreviations

The following abbreviations are used in this manuscript:

WHO	World Health Organization
IM	intestinal microbiota
CNS	central nervous system
ADRC	Alzheimer’s Disease Research Center
POCD	postoperative cognitive dysfunction
SCFA	Short-Chain Fatty Acids
CCK	Cholecystokinin
GLP-1	glucagon-like peptide-1
PYY	peptide YY
BBB	blood–brain barrier
HPA	hypothalamic–pituitary–adrenal
GABA	gamma-aminobutyric acid
MDD	Major Depressive Disorder
fMRI	functional magnetic resonance imaging
BDNF	brain-derived neurotrophic factor
NGP	next generation probiotics
